# Baseline Stiffness Modulates the Non-Linear Response to Stretch of the Extracellular Matrix in Pulmonary Fibrosis

**DOI:** 10.3390/ijms222312928

**Published:** 2021-11-29

**Authors:** Constança Júnior, Maria Narciso, Esther Marhuenda, Isaac Almendros, Ramon Farré, Daniel Navajas, Jorge Otero, Núria Gavara

**Affiliations:** 1 Unitat de Biofísica i Bioenginyeria, Facultat de Medicina i Ciències de la Salut, Universitat de Barcelona, 08036 Barcelona, Spain; cjunior@ibecbarcelona.eu (C.J.); mnarciso@ibecbarcelona.eu (M.N.); marhuenda.esther@gmail.com (E.M.); isaac.almendros@ub.edu (I.A.); rfarre@ub.edu (R.F.); dnavajas@ub.edu (D.N.); jorge.otero@ub.edu (J.O.); 2 The Institute for Bioengineering of Catalonia (IBEC), The Barcelona Institute of Science and Technology (BIST), 08028 Barcelona, Spain; 3 CIBER de Enfermedades Respiratorias, 28029 Madrid, Spain; 4 Institut d’Investigacions Biomèdiques August Pi i Sunyer, 08036 Barcelona, Spain

**Keywords:** extracellular matrix, atomic force microscopy, mechanosensing, fibrosis

## Abstract

Pulmonary fibrosis (PF) is a progressive disease that disrupts the mechanical homeostasis of the lung extracellular matrix (ECM). These effects are particularly relevant in the lung context, given the dynamic nature of cyclic stretch that the ECM is continuously subjected to during breathing. This work uses an in vivo model of pulmonary fibrosis to characterize the macro- and micromechanical properties of lung ECM subjected to stretch. To that aim, we have compared the micromechanical properties of fibrotic ECM in baseline and under stretch conditions, using a novel combination of Atomic Force Microscopy (AFM) and a stretchable membrane-based chip. At the macroscale, fibrotic ECM displayed strain-hardening, with a stiffness one order of magnitude higher than its healthy counterpart. Conversely, at the microscale, we found a switch in the stretch-induced mechanical behaviour of the lung ECM from strain-hardening at physiological ECM stiffnesses to strain-softening at fibrotic ECM stiffnesses. Similarly, we observed solidification of healthy ECM versus fluidization of fibrotic ECM in response to stretch. Our results suggest that the mechanical behaviour of fibrotic ECM under stretch involves a potential built-in mechanotransduction mechanism that may slow down the progression of PF by steering resident fibroblasts away from a pro-fibrotic profile.

## 1. Introduction

Pulmonary fibrosis (PF) is a devastating disease that causes progressive and irreversible destruction of the lung tissue architecture through scaring. Idiopatic pulmonary fibrosis (IPF) is its most severe form, affecting 3 million people worldwide, and with a median life expectancy of 3 years after diagnosis [1]. Besides lung transplantation, there is currently no known treatment for IPF. Recently, two anti-fibrotic drugs, pirfenidone and nintedanib, have been approved to slow down the progression of PF. These drugs are believed to act by inhibiting fibroblast proliferation and differentiation into myofibroblasts, thus likely reducing their ability to modify the composition and architecture of the lung extracellular matrix (ECM) [2]. In this regard, it remains unclear how alterations of the ECM are linked to changes in the mechanical behaviour of the lung in PF and how cells respond to these alterations. However, most importantly, it is not understood whether cyclic stretch further threatens the mechanical homeostasis of the system in PF. Accordingly, further understanding on the role of stretch in the fibrotic lung is needed for the successful development of effective therapies to slow down or stop PF progression [3,4]. There is wide evidence that mechanical alterations of the ECM are correlated with severe respiratory diseases [5]. In the particular case of PF, fibroblasts are highly sensitive to the mechanics of their surroundings, and the presence of aberrant tissue mechanics drives the fibrotic response [6,7,8]. The reparative response of the cells is then dysregulated, resulting in uncontrolled fibroblast proliferation and further deposition of collagenous ECM [9,10]. As a result, the altered mechanical signal hinders normal ECM repair processes, leading instead to abnormal remodelling activity and scaring. There is evidence that this feedback loop is not only a by-product of the disease, but that it actively contributes to the progression of fibrosis [11,12,13,14,15]. Of note, an ECM stiffness threshold value of 3 kPa has been recently identified for murine fibroblasts as tipping point for their fate [12]. In particular, fibroblasts on softer ECM engage an apoptotic program whereas fibroblasts on stiffer ECM become highly proliferative. Nevertheless, this proposed cross-talk between matrix stiffening and fibroblast dysregulation has to be considered in light of the fact that the mechanical properties of the ECM may also be dynamically modulated by cyclic stretch, which is an intrinsic characteristic of lung tissue. Unfortunately, it has not been possible yet to assess how stretch affects the mechanical and viscoelastic response of the fibrotic ECM.

The mechanical properties of the lung can be considered from either a macroscopic or microscopic approach. The macromechanical behaviour of the lung ECM is commonly assessed by subjecting acellular parenchymal strips to uniaxial stretching. The Young’s modulus at the macroscale ($E_{M}$) is then computed as the slope of the stress–strain curve. Such curve typically shows a considerably non-linear behaviour, with stress increasing approximately as an exponential function of strain [16,17,18,19,20]. However, such macroscopic mechanical characterization does not provide direct information on the mechanical microenvironment sensed locally by cells in the lung [21]. Indeed, in a mesh structure such as the lung, macroscopic and microscopic stiffness may not be fully correlated, since the former is mostly determined by the 3D mesh organization of the lung tissue [22,23]. Therefore, a common approach to assess the micromechanical characteristics of the ECM at the cellular scale is based on atomic force microscopy (AFM) [24,25]. AFM measures the mechanical properties of samples at the microscale by indenting them at the nanometer scale and recording the applied force, a strategy referred to as a force–indentation curve. By fitting an appropriate contact model to the force–indentation curve, it is possible to estimate the Young’s Modulus of the ECM sample at the microscale ($E_{m}$). Furthermore, AFM measurements allow also to determine viscoelastic properties. In brief, by applying low-amplitude oscillatory forces at different frequencies, it is possible to estimate the frequency-dependent complex shear modulus of elasticity (G${}^{*}$) [26]. Of note, viscoelasticity is an intrinsic feature of many biological tissues, and its origin has been associated with the major tissue components, namely collagen, elastin and proteoglycans, as well as their crosslinking [27,28]. Accordingly, the use of AFM to assess both the Young’s modulus and viscoelasticity properties of lung ECM at the microscale provides crucial information on the aberrant mechanical environment experienced by resident fibroblasts in PF conditions.

Until recently it was not possible to carry out AFM-based microrheological measurements of biological tissues at different stretch levels, in order to mimic the dynamic environment experienced by the lung ECM. Indeed, we have recently presented a methodology [21] based on a novel polydimethylsiloxane (PDMS) chip that allows us to apply controlled strains to micrometer-thick slices of lung ECM, while performing AFM measurements on them. Accordingly, we can now perform recursive force–indentation and microrheology measurements on the same tissue area and under different levels of controlled tissue stretch. Using this device, we have herein assessed for the first time how physiological levels of equiaxial stretch affect the mechanical properties of lung ECM in fibrosis. In particular, we have focused on rat lungs subjected to bleomycin-induced fibrotic injuries as a model of PF. Interestingly, we identify a switch in the stretch-induced mechanical behaviour of the lung ECM from strain-hardening for healthy ECM to strain-softening for fibrotic ECM. Our findings suggest that breathing-induced ECM stretch leads to a dynamic cyclical decrease in the stiffness of fibrotic ECM. Accordingly, the dynamic mechanotransduction signalling experienced by resident fibroblasts may partially hinder their dysregulation and slow down PF progression.

## 2. Results

### 2.1. Fibrosis Leads to Microscale Changes of ECM Architecture with No Increase in Total Collagen Content

**Figure 1:**
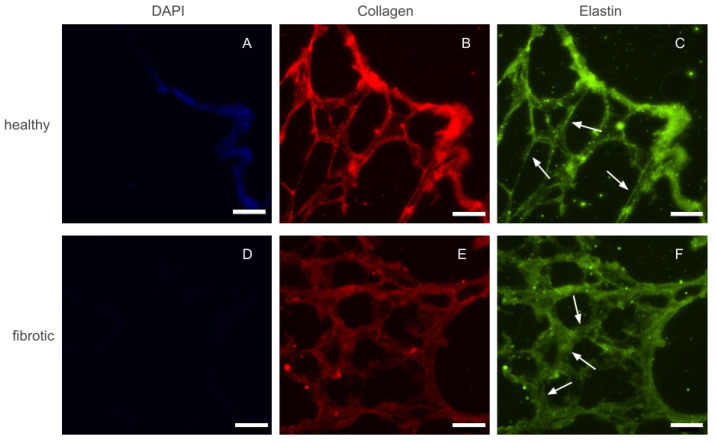
Epifluorescense images of immunostainings performed on a control (top row) and fibrotic (bottom row) groups. DAPI (**A**,**D**) was used to ensure no cellular content was kept after decellularization. Collagen (**B**,**E**) and elastin (**C**,**F**) were stained and are present in both ECMs. Arrows indicate regions with thickened walls in fibrotic samples and normal wall appearance in the healthy counterparts. Scale bar = 50 μm.

After decellularization by perfusion, whole lungs visually appeared well preserved, with their structure maintained. As a whole organ, fibrotic and healthy lungs did not display any marked differences. Before any macro or micro mechanical measurements were carried out, several 20 μm height slices were sectioned and placed on top of positively charged glass slides. Phase contrast imaging of these slices confirmed that the decellularization protocol used throughout this study did not damage the internal microarchitecture of the lung. Similarly, slices of fibrotic lungs displayed localized areas with thickened alveolar walls with respect to healthy ones. However, the size and distribution of the affected areas were highly heterogeneous. Fluorescence images of immunohistochemically-stained lung slices were also collected to assess whether fibrosis lead to marked accumulation of ECM proteins (Figure 1). Collagen and elastin, the two main components of lung ECM, appeared to increase in parallel with the increase of thickness of the alveolar walls. Finally, we took advantage of the fact that slices had also been counterstained with DAPI, to confirm that both healthy and fibrotic lungs had been successfully decellularized and no cellular content remained in the slices.

**Figure 2:**
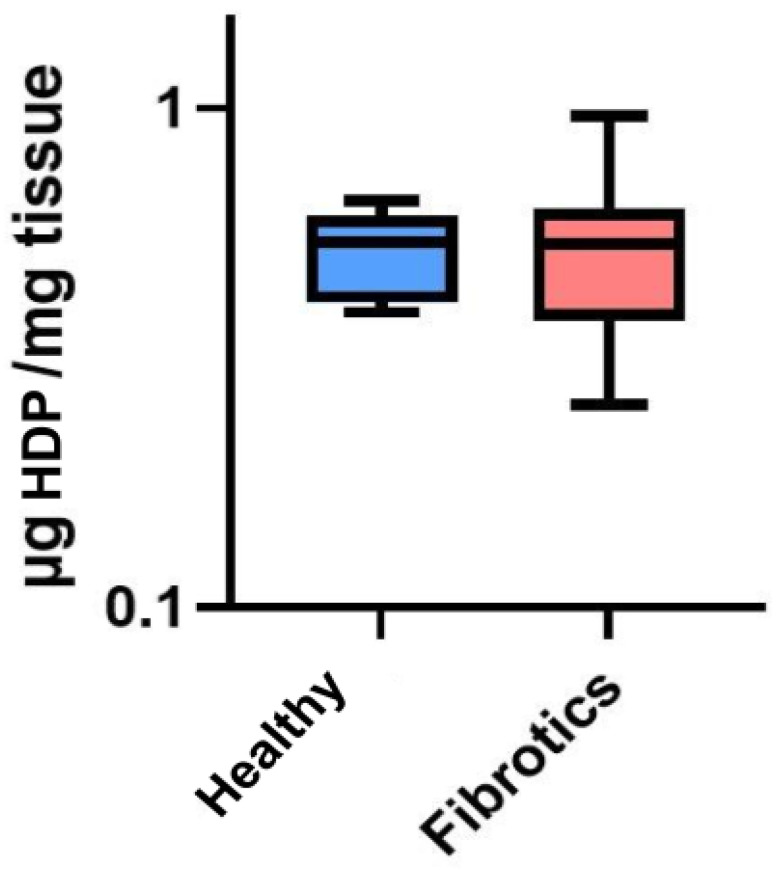
Hydroxyproline (HDP) content in μg per mg of tissue. Data (*N* = 6 for healthy and *N* = 8 for fibrotic lungs) is shown as mean (center line in the box) ± SD (whiskers). There were no differences between groups (p=0.826).

To assess whether the visual changes observed in ECM architecture at the microscale were linked to changes in total collagen content, we performed a collagen content assessment through an hydroxyproline assay (HDP) (Figure 2). No significant change in the total content of HDP was found between healthy and fibrotic lungs (0.52 ± 0.10 μg/mg for healthy vs. 0.54 ± 0.21 μg/mg for fibrotics, p=0.826). Nevertheless, it is relevant to point out that the inter-sample variability was found to be higher in fibrotic samples than in healthy ones (CoV was 39% for fibrotics and 20% for healthy). This result may be linked to our microscale observations on fibrotic lungs, which displayed areas clearly affected by fibrotic scarring in the vicinity of areas with no observable ECM alterations. Together, these results suggest that fibrosis-induced changes of ECM architecture at this point of fibrosis development are mostly observed locally and at the microscale, with a large spatial heterogeneity in the presence of (or its lack thereof) fibrotic scarring. Indeed, this observation is in agreement with results previously reported [14,29,30].

### 2.2. Fibrosis Leads to Macromechanical Stiffening While Non-Linear Elasticity Is Preserved

**Figure 3:**
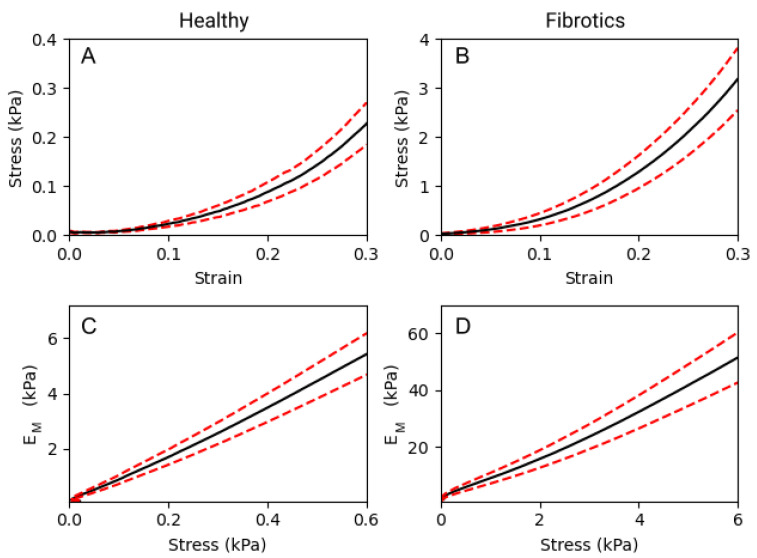
Macromechanics of decellularized lung strips subjected to uniaxial tensile testing. (**A**,**B**) represent the stress–strain relationship of healthy lung ECM strips and fibrotic strips, respectively. (**C**,**D**) show the dependence of the $E_{M}$ with stress, on healthy and fibrotic samples, respectively. Data (*N* = 6 for healthy and *N* = 8 for fibrotic lungs) is shown as mean (black solid lines) ± SE (dashed red lines).

To compare the macromechanical properties of fibrotic and healthy decellularized lungs, tensile tests were performed. As expected, a strong non-linear relationship between stress and strain was observed in both groups (Figure 3A,B). In detail, for any given value of strain, the elastic modulus ($E_{M}$) obtained for fibrotic samples was approximately one order of magnitude larger than that obtained for healthy ones. If we focus on three specific strain levels applied to the lung strips (0.1, 0.2 and 0.3 strain), we report that $E_{M}$ values were 0.33 ± 0.07 kPa, 0.80 ± 0.14 kPa and 1.93 ± 0.31 kPa, respectively, for healthy samples. For the fibrotic samples, the results obtained were of 4.67 ± 1.05 kPa, 10.83 ± 2.22 kPa and 25.09 ± 4.67 kPa, respectively. Therefore, $E_{M}$ was observed to increase linearly with stress both in healthy and fibrotic samples (Figure 3C,D). According to the Fung’s model [17], when $E_{M}$ follows a linear increase with stress, it is valid to use an exponential approximation fit to the stress–strain curves, from which $E_{M}\propto e^{\alpha \epsilon }$. Using this model, we obtain a nonlinearity index α = 8.86 ± 0.02 for the healthy and α = 8.41 ± 0.03 for the fibrotic samples. Both indices are very similar indicating that while fibrosis broadly stiffens pulmonary tissue at the macroscale, it does not alter the characteristic nonlinear behaviour displayed by its stress–strain curves.

### 2.3. At the Microscale, the Tendency of Lung ECM to Stiffen with Stretch Strongly Depends on Its Baseline Stiffness

**Figure 4:**
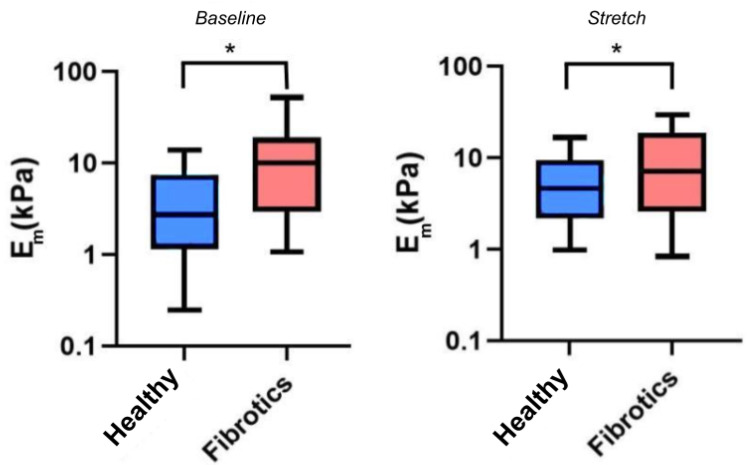
Young’s Modulus $E_{m}$ measured by AFM. Boxplots represent the distribution of the data by $E_{baseline}$ (**left**) and $E_{stretch}$ (**right**). The center line in the boxplots represents the median value. (* p<0.05).

Having confirmed that fibrosis lead to mechanical changes at the macroscale, we next sought to verify whether mechanical changes would also be manifest at the microscale, and whether stretch would further affect these mechanical alterations. For these experiments, we used AFM to probe the local micromechanical stiffness, as assessed by the microscale Young’s modulus $E_{m}$ (Figure 4). Accordingly, for the remaining of this report we refer to $E_{baseline}$ as the microscale Young’s modulus under unstretched baseline conditions, and to $E_{stretch}$ as the microscale Young’s modulus under stretch conditions. In our results we found that the median value for $E_{baseline}$ was 2.7 kPa (Q1–Q3 being 1.3–7.1 kPa) for the healthy samples, and 10.1 kPa (Q1–Q3 being 3.4–17.7 kPa) for the fibrotic ones, thus confirming a stiffening of lung ECM due to fibrosis at the microscale (p<0.05). Similarly, the median values for $E_{stretch}$ were 4.7 kPa (Q1–Q3 being 2.3–8.6 kPa) for healthy, and 6.6 kPa (Q1–Q3 being 2.8–18.7 kPa) for the fibrotic ones. On the one hand, these particular set of results show that, on average, fibrotic samples are still stiffer than the healthy ones when subjected to stretch (p<0.05). However, surprisingly, these results also show that the response to stretch diverged between health and fibrotic samples. In particular, the healthy ECM samples displayed a marked strain-hardening (2-fold increase in $E_{m}$) whereas the fibrotic samples displayed marked strain-softening (40% decrease in $E_{m}$) due to stretch. However, no statistical significance was reached (p=0.062) regarding the interaction between groups (healthy or fibrotics) and state (baseline or stretch).

**Figure 5:**
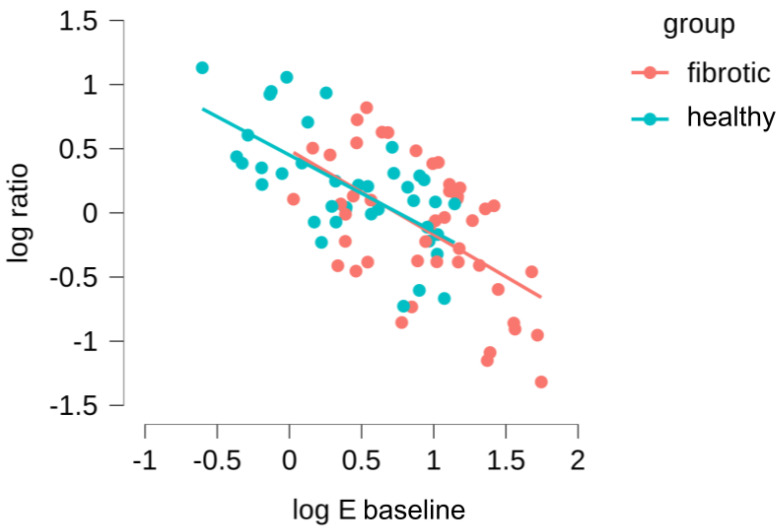
Dependence of the ratio ($E_{stretch}$/$E_{baseline}$) on the Young’s modulus ($E_{m}$) measured in the initial baseline state. Red data points account for measurements performed in bleomycin-treated lungs and blue points for the healthy lungs.

Subsequently, we took advantage of the fact that our measurements had been carried out in the same ECM location before and during stretch to further refine our data analysis. First, we computed for each probed region a new parameter, the ratio of microscale Young’s moduli, as $ratio=E_{stretch}/E_{baseline}$. Thus, ratio < 1 indicates strain softening and ratio > 1 indicates strain hardening. Then, we assessed whether there was a correlation between baseline stiffness ($E_{baseline}$) and the tendency to strain-harden or strain-stiffen (ratio), both for the healthy and fibrotic samples (Figure 5). We found a relationship that follows a strong log-log behavior, and what is more interesting, there is a total overlap in this behavior for datapoints belonging to healthy and fibrotic samples. This was verified with an ANCOVA test, confirming that there were no significant differences between the log-log relationships for the healthy and fibrotic datapoints (p=0.979). Accordingly, we then combined all the datapoints for the healthy and fibrotic samples, fitting a power law relationship to the data which yields $ratio=2.904\times {E_{baseline}}^{\left(-0.629\right)}$ (Pearson’s *r* of −0.648 and p<0.001). We can use this expression to identify a threshold value of $E_{baseline}$ (subsequently defined as $E_{thresh}$) for which the parameter ratio is 1, and thus the system experiences a switch in mechanical behavior from strain-hardening to strain-softening. The value we obtained for $E_{thresh}$ is 5.4 ± 1.1 kPa, which is in the range of $E_{baseline}$ for healthy lungs and much lower than the values of $E_{baseline}$ for fibrotic lungs. Together, these finding suggests that the mechanical response of lung ECM to stretch is not fundamentally altered due to fibrosis. On the contrary, fibrosis simply offsets $E_{baseline}$ towards higher values, well above the computed $E_{thresh}$ of the lung ECM system. As a consequence, the fibrotic lung is found within the mechanical regime that leads to strain-softening while its healthy counterpart remains within the mechanical regime that displays strain-hardening.

### 2.4. Fibrotic Lungs Tend to Fluidize with Stretch However, Healthy Counterparts Tend to Solidify

**Figure 6:**
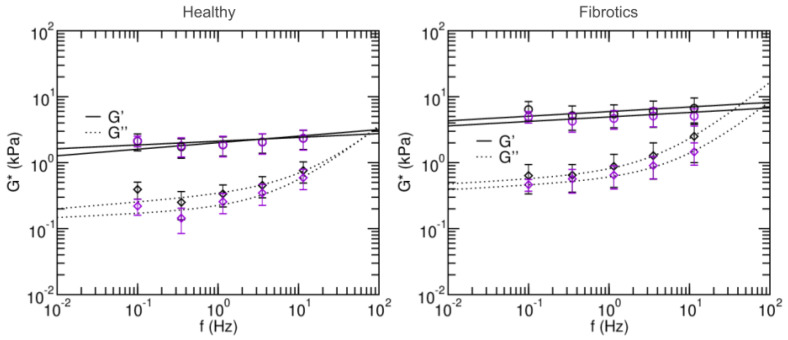
Frequency-dependent shear elastic moduli of fibrotic lungs (**left panel**) and healthy lungs (**right panel**). Solid and dashed lines are fits of the two-power law model. Black and purple circles are measurements done in baseline and stretched states, respectively. Data are mean ± SE (*N* = 14). Fit parameters are shown in Table 1.

To further characterize the microscale mechanical properties of fibrotic lungs and their response to stretch, we next focused on their viscoelastic behavior, and we used AFM to measure the microrheological response of the lung slides to different probing frequencies (Figure 6). Broadly, the values found for storage modulus G${}^{\prime }$ and loss modulus G${}^{\prime \prime }$ were again larger for fibrotic samples than for healthy ones. Similarly, stretch lead to decreases in G${}^{\prime }$ and G${}^{\prime \prime }$ for fibrotic samples and no remarkable changes in the healthy. Specifically, the storage modulus G${}^{\prime }$ of the fibrotic samples at 0.1 Hz was 6.40 ± 2.03 kPa in baseline and 4.96 ± 0.98 kPa in stretch. For the healthy these were 2.12 ± 0.61 kPa in baseline and 2.14 ± 0.39 kPa in stretch. In all cases, G${}^{\prime \prime }$ at low frequencies was an order of magnitude smaller than its corresponding G${}^{\prime }$. As expected, at frequencies higher than 1 Hz, G${}^{\prime \prime }$ becomes more prominent. In both healthy and fibrotic samples, G${}^{\prime \prime }$ decreased with applied stretch.

Microrheology data was then fitted to a two-power law model to further characterize the fluid-like or solid-like behavior of lung ECM (Equation (12)). Firstly, coefficients A and B in the model can be interpreted as an index of stiffness at the low- and high- frequencies, respectively. Both were higher in the fits obtained for the fibrotic lungs vs. healthy (p<0.05), again confirming that fibrosis stiffens lung ECM. Based on the two-power law model, we can also define a transition frequency ($f_{t}$) as the frequency at which $A{\left(if_{t}\right)}^{\alpha }=B{\left(if_{t}\right)}^{\beta }$. This frequency defines the transition from which the material goes from being dominated by the storage modulus (more solid-like) to being dominated by the loss modulus (more fluid-like). The transition frequency was found to be higher in healthy than in fibrotic measurements in the baseline state, but the opposite was found in stretch conditions. Similarly, for the low frequency α index, it was found to decrease with stretch for the healthy lungs, and increased with stretch for fibrotic lungs. Together, these observations suggest that in the healthy lungs, stretch induces a solidification of the matrix, whereas for fibrotic lungs, stretch induces a fluidization of their matrix.

## 3. Discussion

The mechanical properties of lung parenchyma are frequently measured via tensile tests that use small strips and apply uniaxial stretch onto them [18,21,32]. Importantly, the stiffness of the air-filled lung is determined by the surface forces within the alveoli and the elastic forces of the ECM [33]. Nevertheless, when submersed in liquid during the tensile test procedure, the lung strips lose the air-liquid interface surface forces. Furthermore, the results obtained through tensile tests do not report solely the direct contribution of the intrinsic micromechanical features of the acellular scaffold, but are also modulated by the 3D network architecture of the alveoli [21]. Thus, the information gathered through macroscale tensile tests is not best suited to fully characterize the actual lung micromechanical environment and the mechanosensing events it may trigger at the cellular level. In this regard, our results for Young’s modulus obtained at the microscale are larger than those we obtain at the macroscale, both for healthy and fibrotic samples and in agreement with our previous studies [21].Furthermore, as fibrosis is known to be heterogeneously spread throughout tissue, local mechanical changes and their contribution to the overall tissue mechanics might be overlooked by performing organ-scale measurements. Indeed, it has been reported that the most common type of lung fibrosis is driven by increased tension at the alveolar level, thus further stressing the need to characterize the mechanical environment at the microscopic cellular scale [14]. Lung tissues are subjected to strain levels in the order of 10–20% during normal breathing [33,34]. Accordingly, our PDMS chips were stretched at ≈15% equiaxial stretch to mimick the physiological strain range. Nevertheless, since our lung slices are adhered to the deformable membrane, the ECM is subjected to 2D strain. Conversely, inflation of the native lung is 3-dimensional, and can result in the partial folding-unfolding of the alveolar septa at low volumes [33]. As a result, it may be difficult to accurately relate the 2D strain values of our measurements to 3D volume changes in the native lung.

The bleomycin experimental model is the most employed and characterized experimental model of fibrosis in rat. In our experiments, we chose to use 2 U/kg because higher doses (<3 U/kg) can cause significant mortality in animals. One commonly stated limitation of the bleomycin model is that the fibrosis is not progressive and in fact, resolves with time. In particular, the response to bleomycin occurrs in three distinct phases: inflammation phase (days 1–2), active fibrosis phase (days 7–14), and late fibrosis (days 21–35). In our experiments, we chose to focus on the active fibrosis phase (around 10 days after bleomycin infusion), where increased levels of pro-fibrotic mediators associated with increased matrix deposition in the lung have been reported by others [35,36] Nevertheless, a previous study on collagen content on a bleomycin model of pulmonary fibrosis indicates that the collagen content only increases significantly 28 days after the injection [37]. Furthermore, a recent work reported by Song and colleagues suggests that the use of HDP content assay to evaluate the severity of fibrosis in the bleomycin-induced model in mice highly depends on the status of native collagen. It showed that a marked increase on HDP content is observed 4 weeks after the bleomycin treatment, and that it sustained for further 10 to 16 weeks after treatment [30]. Given that animals in our model were sacrificed 10 days after the injection of bleomycin, our findings suggest that mechanical alterations take place in the ECM before the aberrant deposition of collagen starts. It has been suggested that the increased stiffness seen in pulmonary fibrosis is a function of dysregulated post-translational collagen cross-linking rather than collagen concentration increase [29]. Similarly, a study on liver fibrosis suggest that the increase in stiffness takes place before or without measurable increases in collagen content [38]. Our findings thus align with previous reports not only in pulmonary but also in other types of fibrosis, further confirming that up to one order of magnitude difference in stiffness between healthy and fibrotic samples can be observed by tensile tests even though no increase in collagen content is found.

The spatial distribution of lung fibrosis is highly heterogeneous, typically resulting in a high dispersion of measured values of the Young’s Modulus when using AFM [11,39]. This fact may introduce drawbacks to the way the statistics of local AFM measurements are carried out, as the degree of scarring can vary largely between and within animals. Thus, simply averaging all the measured Young’s modulus values may make it difficult to reach a strong statistical result in the context of lung fibrosis and stretch. With this in mind, we moved beyond data pooling (as shown in Figure 4) and aimed at assessing our results at the single location level (as shown instead in Figure 5) comparing the individual $E_{m}$ values measured before and during stretch. With this approach, we are able to find a strong relationship between the local initial stiffness and the tendency of the tissue to locally stiffen or soften due to stretch. Furthermore, this approach has allowed us to dissect out the contribution of fibrosis to lung ECM under stretch. Of note, local fibrosis induces only an offset in the initial mechanical state of the lung, which tends to displace the ECM well into the strain-softening regime. Nevertheless, the fact that both datapoint distributions for baseline and stretch measurements align in Figure 5 strongly suggest that the mechanical effect of fibrosis on the ECM is not due to a fundamental change in the nature of its fibrillar composition or architecture at the nanoscale, but it is rather a local microscale buildup and reorganization of the existing matrix.

It has been previously reported [5] that the ECM can be modeled as a fiber network. As fibrosis is characterized by dysregulated collagen fiber production, and assuming that the crosslinking of the fibers is compromised in the reparatory response of the tissue, this situation would potentially translate into a matrix with several non-crosslinked or weakly-crosslinked networks. Recent modelling work [40] suggests that fiber entanglements found in such weakly-crosslinked tissues materials produce a self-balanced stress in response to tension that leads to increases in stiffness only up to a point. Beyond that strain threshold, the matrix softens due to fiber separation. This prediction is reminiscent of our findings, but we emphasize that, in our experiments, strain levels were kept constant at 15% for all samples probed. Accordingly, we did not observe a strain threshold beyond which samples displayed strain-softening, but rather we identified a stiffness threshold (likely linked to the degree of weak crosslinks found in the network locally) beyond which the sample would similarly display strain-softening.

Microrheology experiments allow us to not only measure the stiffness of tissues, but also to characterize their tendency to behave as solid-like or liquid-like materials in response to a load. In general, when strained or sheared, materials that display strain-hardening also display solidification, whereas strain-softening is accompanied by a fluidization [41]. Indeed, such is also our observation for lung ECM, both for healthy and fibrotic samples. Accordingly, we hypothesize that the $E_{thresh}$ value we obtain as threshold stiffness between strain-hardening behavior and strain-softening behavior can also be used to predict stretch-induced solidification versus fluidization. Of note, human lung epithelial cells have been shown to display mild solidification when subjected to stretch [42], thus showing a parallel behavior to that displayed by healthy lung ECM. It remains nevertheless unclear what is the viscoelastic and fluid- vs. solid-like behavior in response to stretch in other cell types more prevalently involved in PF, i.e., lung fibroblasts, and whether their activation state modulates that response.

The mechanical properties of the ECM are relevant in modulating correct cellular activity, and they become particularly crucial when it comes to the reparative responses to tissular damage. In the case of fibrosis, its progression is driven by a runaway reparative feedback loop. Accordingly, it has been suggested that an intrinsic change in ECM’s viscoelasticity may be instrumental in initiating such feedback loop, which maintains in an activated state the cells responsible for the reparative response, and leads to the aberrant formation of scar tissue [37]. Of note, independent studies on fibroblast response to substrate stiffening or to stretch revealed that these are crucial factors that determine the progression of PF [13,14]. Importantly, Liu and colleagues [12] showed recently that substrate stiffening determines whether fibroblasts will transition to an apoptotic or proliferative state after their initial activation. The threshold value they identified for mice fibroblasts was in the pathological range of stiffness for murine lung ECM, namely 3 kPa, with fibroblasts on softer substrates engaging an apoptotic response whereas fibroblasts on stiffer substrates becoming highly proliferative. Interestingly, this threshold stiffness value is in the range of the one reported in our study here, where we report a value of 5.4 kPa as the transition point from strain-hardening to strain-softening of lung ECM in response to stretch. Combining these findings, we can hypothesize that the effect of breathing-induced alveoli stretch would cyclically decrease fibrotic ECM stiffness, thus shifting it away from a mechanical environment that would otherwise further promote fibroblasts proliferation and PF progression. Together, these results suggest an unreported mechanical mechanism the can be determinant in the modulation of fibroblast fate, and may constitute a built-in attempt of the system to slow down the progression towards a pro-fibrotic phenotype in fibroblasts.

From a clinical perspective, it has been shown that exercise reduces lung fibrosis in rodents with already established ECM fibrotic alterations by bleomycin infusion [43]. Similarly, there is preliminary evidence that exercise and pulmonary rehabilitation may be positive for patients with lung fibrosis [44,45]. Nevertheless, it is unknown whether such positive effects of exercise in experimental and clinical lung fibrosis are caused by the reparative events elicited by exercise (e.g., in the inflammatory cascade) or by the increased lung stretching associated with exercise. However, it is interesting to note that a recent clinical trial in patients with lung fibrosis has shown that an exercise program involving only the lung (based exclusively on deep breath exercises) improved the pulmonary function and quality of life of patients [46]. All these indirect data are concurrent with our findings here that stretch induces strain-softening in fibrotic ECM, which could thus promote both restoration of lung mechanics and fibroblast reprogramming. The results reported here thus allow to draw a new relationship between lung ECM stiffness, fibroblast mechanosensing and fibrosis progression, with promising applications in tissue engineering, clinical therapies and disease modeling.

To conclude, we identify a switch around 5 kPa in the lung ECM’s mechanical response to stretch, from strain-hardening to strain-softening. In brief, the stiffer fibrotic lung ECM is found within the mechanical regime that leads to strain-softening while its healthy counterpart remains within the mechanical regime that displays strain-hardening. As a result of those stretch-induced changes in ECM mechanics, breathing-induced alveoli distention would cyclically shift fibroblasts away from their pro-fibrotic phenotype, thus hindering PF progression. Our basic research results are in line with recent clinical findings suggesting that a regime of deep breath exercises may improve the pulmonary function of PF patients.

## 4. Materials and Methods

### 4.1. In Vivo Model of Pulmonary Fibrosis

The study was carried out in lungs excised from *N* = 14 adult male Sprague-Dawley rats (≈8 weeks old, 300 g) (Charles River, MA, USA). The experimental procedure was approved by the Ethical Committee for Animal Experimentation of the University of Barcelona following local and European regulations. Animals were divided into two groups: a healthy control group (healthy, *N* = 6) and a group with induced lung fibrosis (fibrotics, *N* = 8). Fibrosis was induced by an intratracheal bleomycin infusion model, as described elsewhere [47]. Briefly, rats were anesthetized (isofluorane inhalation), followed by an infusion of bleomycin (2 U/kg; Sigma-Aldrich, St. Louis, MO, USA) diluted in 75 μL of phosphate-buffered saline (PBS) into the tracheal lumen. To assess the effectiveness of the bleomycin response, we monitored the animals’ weight until sacrifice. As expected, in the inflammatory acute phase (1–3 days post injection) rats treated with bleomycin lost weight. After 7 days, they started to recover weight thus confirming the active fibrosis phase. All animals were cared for under normal living conditions for 10 days, when they were anesthetized with urethane and sacrificed by exsanguination. Lungs were extracted en bloc with the heart, pulmonary artery and trachea, and stored at −80 °C until the decellularization procedure was carried out. The healthy group followed an identical procedure, but only PBS (vehicle) was intratracheally infused.

### 4.2. Lung Decellularization

Excised lungs were decellularized following a protocol previously described [21]. In brief, after thawing at room temperature (RT) lungs underwent a sequential perfusion of decellularizing and washing solutions through the left ventricle of the heart and trachea simultaneously. They were firstly washed with phosphate buffered saline (PBS) (ThermoFisher Scientific, Waltham, MA, USA) for 15 min and with deionized water for 10 min to induce cell osmotic shock. Afterwards, 0.1% Triton X-100 and 2% sodium deoxycholate (SDC) (Sigma-Aldrich, St. Louis, MO, USA) solutions were perfused for 30 min and 2 h, respectively, followed by perfusion of deionized water for 15 min. In a final step, perfusion for 15 min of NaCl (Sigma-Aldrich, St. Louis, MO, USA) solution followed by 15 min of PBS was used to obtain the final decellularized lung. For all decellularized lungs, the right upper lobe was excised and stored at −80 °C. This part of the lung was later used for tensile testing purposes, The remainder of the decellularized lung was bronchially perfused and embedded in optimal cutting tissue medium (OCT compound, Sakura, CA, USA) and stored at −80 °C. This part of the lung was later used for AFM measurements and immunohistochemical imaging.

### 4.3. Immunohistochemical Imaging

To investigate the structural characteristics of the ECM, fluorescent immunostaining of decellularized lung slices was performed. Previously stored slides were left to thaw and rinsed with PBS to remove the OCT. Then, samples were fixed using paraformaldehyde (PFA) 4% for 15 min at RT and washed 3 times with PBS for 5 min each. Samples were then blocked with a solution of 10% fetal bovine serum (FBS) for 30 min at RT. Samples were incubated overnight at 4 °C with primary antibodies against elastin (goat anti-elastin, 1:50, Santa Cruz, Dallas, TX, USA) and collagen I (rabbit anti-collagen I, 1:300, Abcam, Cambridge, MA, USA). Secondary antibodies (goat anti- rabbit Alexa Fluor 647, 1:200 Thermo Fischer and donkey anti-goat, Alexa Fluor 488, 1:200, Thermo Fischer) were added and left for 2 h at 37 °C. Nuclear counterstain DAPI (Hoechst 33342, Thermo Fischer Scientific, Waltham, MA, USA) was then added and left for 45 min at RT, and rinsed 3 times with PBS for 5 min. The slides were carefully dried and mounted using Fluoromount mounting media (Thermo 295 Fisher Scientific, Waltham, MA, USA). Epifluorescense images were acquired with a Leica SP5 inverted microscope equipped with a cooled CCD camera (C9100, Hamamatsu Photonics K.K., Hamamatsu, Japan) using a 20XPlan Fluor objective (Nikon, Tokyo, Japan).

### 4.4. Collagen Content Assessment

A colorimetric hydroxyproline (HDP) assay kit (MAK008, Sigma Aldrich, Darmstadt, Germany) was used to estimate the collagen content of the samples that had been previously used for the macromechanical measurements. Briefly, the tissue samples (*N* = 14) were hydrolyzed in HCl at 120 °C for 3 h, and processed according to the manufacturer’s instructions. The total HDP concentration is determined by the reaction of oxidized HDP with 4-(Dimethylamino)benzaldehyde (DMAB), which results in a colorimetric product, proportional to the HDP present. The final absorbance was measured at 560 nm with a microplate spectrophotometer (Multiskan FC, Thermo Fisher Scientific).

### 4.5. Measurement of Macroscale Mechanics by Tensile Stretching

Assessment of the macromechanical properties of the strips was performed as described elsewhere [48]. Briefly, to compute the macroscopic Young’s modulus ($E_{M}$) the following modelling procedure is used. First, we consider that a tissue strip subjected to a tensile force (*F*) experiences a stretch ratio (λ) given by:

(1) $$\lambda =\frac{L}{L_{0}}$$

where *L* is the strip’s length along the direction of *F* and $L_{0}$ the strip resting length defined as its length measured for an applied force of 0.1 mN. The Lagrangian strain (ϵ) and Lagrangian tensile stress (σ) are then defined as:

(2) ϵ=λ−1

(3) $$\sigma =\frac{F}{A_{0}}$$

where $A_{0}$ is the cross-sectional area of the strip in the undeformed state, and which based on geometrical factors of the strip can be computed as:

(4) $$A_{0}=\frac{M}{\rho L_{0}}$$

where *M* is the mass of the strip and ρ its estimated density (assumed to be 1.06 g/cm${}^{3}$) Finally, the macroscopic Young’s modulus $E_{M}$ is calculated as the derivative along the σ-ϵ curve:

(5) $$E_{M}=\frac{d\sigma }{d\epsilon }$$

The experimental procedure (tensile stretch testing) to measure the macroscopic Young’s modulus $E_{M}$ based on the modelling above is as follows. Firstly, decellularized lung samples were thawed at 4 °C overnight. Strips (≈7 × 2 × 2 mm) were cut using a scalpel, gently dried with tissue paper and its mass was determined using a precision scale. The end of each strip was firmly fixed to a hook using cyanoacrylate glue. One of the hooks was placed on the lever arm of a servo-controlled displacement actuator (300C-LR, Aurora Scientific, Aurora, ON, Canada) and the other was placed on a force transducer (404A, Aurora Scientific, Aurora, ON, Canada). The force transducer enabled measurement of the force applied to the strip with a resolution of 2 μN. All tensile stretch testing experiments of decellularized lung samples were performed in PBS at 37 °C. Before carrying out the measurement, the strips were preconditioned by applying 10 cycles of stretch at 0.2 Hz and maximum strain of ≈30%. After returning to the relaxed state, data from 10 cycles was collected. The first cycle was discarded, and a final mean σ-ϵ curve was obtained by averaging the 9 remaining individual σ-ϵ curves. Following Equation (5) above, the macroscopic Young’s modulus was computed along the mean σ-ϵ curve as the local derivative of an exponential function, using a 0.05 strain fitting window.

### 4.6. Polydimethylsiloxane (PDMS) Chip Fabrication

The chips used to stretch ECM thin sections for AFM measurements were fabricated using a protocol previously described [21]. In brief, the protocol involves parallel fabrication of a PDMS block as well as a thin stretchable membrane that will act as the block’s substrate. To produce the PDMS block, a 3D-printed (Ultimaker 2, Utrecht, The Netherlands) negative mold was attached to a 100 mm culture dish, and a 10:1 PDMS mixture of pre-polymer and curing agent (Sylgard 184 kit, Dow Corning, Midland, MI, USA) was poured into it and left to be cured for 2 h at 65 °C. To produce the thin stretchable membrane, a glass slide of 76 mm × 26 mm (Deltalab, Barcelona, Spain) was sequentially immersed and slightly agitated in acetone, methanol and isopropyl alcohol and submitted to oxygen plasma treatment using a plasma cleaner (PDC-002, Harrick Scientific Products Inc, Pleasantville, NY, USA) at 18 W for 30 s. The slide was then treated for 1 h with Repel Silane [(Tridecafluoro-1,1,2,2-tetrahydrooctyl) trichlorosilane] (ABCR GmbH & Co. KG, Karlsruhe, Germany) and PDMS was poured onto the treated glass slide. The slide was spun (Laurell Technologies Corporation, North Wales, PA, USA) at 1000 RPM for 60 s to obtain a membrane with a thickness of ≈20 μm. To assemble the chip, the surfaces of the thin membrane and of the PDMS block were both activated with a portable corona treater (Electro Technic Products, Chicago, IL, USA) for 1 min, and immediately pressed against each other to obtain a firm bond. To finalize chip assembly, a 0.3 mm ID silicone tube was inserted in the vacuum chamber of the chip, and any possible leaks were sealed with PDMS mixture and cured. The assembled chip was then attached to the bottom of a 35 mm culture dish and stored.

### 4.7. Measurement of Microscale Mechanics by AFM

Sections of frozen decellularized lungs were thinly cut using a cryostat (Leica CM 3050 S, Leica) to obtain slices of ≈20 μm thickness. For slicing, sections comprised of subpleural regions were chosen to exclude the presence of large airways. To adhere the obtained thin lung slices onto the previously assembled chip, the chip’s top membrane was pre-treated with (3- Aminopropyl) triethoxysilane (APTES, Sigma-Aldrich, St. Louis, MO, USA) 10% in absolute ethanol (*v*/*v*) for 1 h at RT, followed by genipin 1.5% in PBS (*v*/*v*) for 30 min at RT. The slices were carefully laid on the chips’ top surface and then left overnight at 37 °C and 100% humidity to promote firm adhesion of the slice to the membrane. For AFM measurements, a chip with a previously attached sample was placed on the stage of an inverted optical microscope (Nikon TE-2000, Tokyo, Japan) equipped with a CCD camera (Marlin F145B2, Allied Vision Technologies, Stadtroda, Germany) and a custom-built AFM. The inlet tube of the chip was connected to a servo-controlled vacuum pump, which allowed the controlled application of negative pressures and consequently lead to the lung slice being stretched equiaxially.

Measurements were made with the lung slice submerged in PBS at 37 °C. Cantilevers used were SPC-200804-0,1 with a silicon nitride 11 μm-diameter sphere attached to their end (Bruker, CA, USA). Cantilevers had been individually calibrated by the manufacturer using an interferometer, and displayed spring constants (*k*) of 0.161 N/m and 0.170 N/m. In our custom-built AFM system, the piezoactuators are coupled to strain gauge sensors (Physik Instrumente, Karlsruhe, Germany), to enable the 3D displacement of the cantilever, as well as the precise measurement of its vertical displacement (*z*). The deflection of the cantilever (*d*) is assessed via a quadrant photodiode (S4349, Hamamatsu, Hamamatsu City, Japan). For each probed lung slice, a deflection-displacement (*d*-*z*) curve was initially obtained on a bare region of the culture dish, and the slope of the curve was calculated to calibrate the relationship between the cantilever deflection and the photodiode signal. A linear calibration curve with a sharp contact point evidenced a clean and undamaged tip. The following protocol combining sequential force–indentation and microrheology measurements was then used on multiple locations of each sample, both before and under stretch conditions. In brief, for each probed location, 10 (*d*-*z*) curves were recorded consecutively with a ramp amplitude of 5 μm and a frequency of 1 Hz (tip velocity of 10 μm/s). After the (*d*-*z*) curves had been acquired, the tip was set at an operating indentation of ≈500 nm and a multifrequency oscillation signal was applied. This signal was composed of 5 sine waves (0.1, 0.35, 1.15, 3.55, 11.45 Hz). To avoid harmonic cross-talk, the frequency of the sinusoidal components were non-sum and non-difference between each other. Each multifrequency acquisition had a duration of 140 s. Once the combined (*d*-*z*) curves and microheology measurements had been carried out in a given sample location, the cantilever was moved approximately 100 μm apart and another set of measurements was carried out, for a total of 4 sample locations measured for each lung slice. Subsequently, a ≈15% equiaxial stretch was applied to the sample and the whole protocol was repeated as described above. Optical imaging of the lung slice was used to make sure the AFM tip was approximately re-positioned in the same 4 sample locations before and during stretch. Furthermore, the level of stretch applied for each sample-chip combination was confirmed using again optical means, by computing the relative change in distance between two points of the ECM images recorded before and during stretch application. Experiments where the applied stretch levels measured experimentally were lower than 13% or higher than 17% were discarded.Based on our experimental conditions, which consider a sample indentation of 0.5 μm and a 11 μm diameter tip, the diameter of the effective contact surface for our measurements is estimated to be 4.7 μm. Taking this into account, measurements were performed only in areas where the sample had a width larger than approximately 30 μm to fulfill the assumptions of the Hertz’s model for contact mechanics.

### 4.8. AFM Data Processing

To compute the microscale Young’s modulus from the obtained (*d*-*z*) curves, we used the following approach based on Hertz’s model. Firstly, to compute the force (*F*) applied by the cantilever, the following relation is used:

(6) $$F=k\times (d-d_{0})$$

and the indentation (δ) is calculated from:

(7) $$\delta =\left(z-z_{0}\right)-\left(d-d_{0}\right)$$

where $d_{0}$ is the deflection offset and $z_{0}$ the cantilever displacement at the tip-sample contact point. To analyze the *F*-δ curves, the Hertz model for a sphere indenting a semi-infinite half-space is used:

(8) $$F=\frac{4E_{m}}{3(1-\upsilon^{2})}R^{\frac{1}{2}}\delta^{\frac{3}{2}}$$

where *R* is the radius of the sphere and υ is the Poisson’s ratio. The former is assumed to be 0.5. Expressing Equation (8) in terms of *z* and *d* the following relationship is obtained:

(9) $$d=d_{0}+\frac{4E_{m}}{3k(1-\upsilon^{2})}R^{\frac{1}{2}}{\left[\left(z-z_{0}\right)-\left(d-d_{0}\right)\right]}^{\frac{3}{2}}$$

For each obtained *d*-*z* curve, the parameters $E_{m}$, deflection offset ($d_{0}$) and displacement at the tip-sample contact point ($z_{0}$) are simultaneously calculated by non-linear least-squares fitting using custom-developed scripts (MATLAB, The MathWorks Inc., MA). Only the datapoints of the approaching curve up to a maximum indentation of ≈500 nm are used for the fitting procedure.

For microrheology measurements at low-amplitude oscillations around $\delta_{0}$, Equation (8) can be expressed in terms of the shear modulus of elasticity $G=E_{m}/\left[2\left(1+\upsilon \right]\right)$, so that the complex shear modulus ($G^{*}$) is then expressed in the frequency domain as:

(10) $$G^{*}\left(\omega \right)=\frac{\left(1-\nu \right)}{4{\left(R\delta_{0}\right)}^{1/2}\tan\theta }\left[\frac{F(\omega )}{\delta (\omega )}-i\omega b\left(0\right)\right]$$

where ω is the angular frequency (ω=2πf), and F(ω) and δ(ω) are the Fourier transforms of *F* and δ at ω, respectively. The iωb(0) term is the viscous drag hydrodynamic force correction [49]. $G^{*}$ is represented in terms of its real part G${}^{\prime }$, corresponding to the storage modulus, and its imaginary part G${}^{\prime \prime }$, corresponding to the loss modulus. The storage modulus accounts for the elastic energy stored, and the loss modulus for the energy dissipated. From these terms the loss tangent can be calculated as:

(11) $$\eta =\frac{G^{{}^{\prime \prime }}}{G^{{}^{\prime }}}$$

which is an index of the solid-like (<1) or liquid-like (>1) behaviour of the sample.

The computed $G^{*}\left(\omega \right)$ data can be further modeled to a linear superposition of two power laws as:

(12) $$G^{*}\left(\omega \right)=A{\left(i\omega \right)}^{\alpha }+B{\left(i\omega \right)}^{\beta }$$

The first term of this model assumes a low-frequency regime characterized by a weak power law, and the second term accounts for the high-frequency regime. The exponent is related to the loss tangent as η=tan(απ/2), and can be interpreted as a measure of the proximity to a pure solid-like (α = 0, G${}^{\prime \prime }$ = 0) or pure fluid-like behaviour (α = 1, G${}^{\prime }$ = 0). In our current analysis, the β exponent was imposed to be 3/4 [31]. Model fits were performed using the open-access user-friendly online platform fitteia [50] (fitteia.org, accessed on 28 November 2021) that uses the non-linear least-squares minimization method with a global minimum target, provided by the numerical routine MINUIT from the CERN library [51].

### 4.9. Statistics

Unless stated otherwise, data is presented as mean ± SE. Statistical analysis was performed with JASP (JASP Team (2020), JASP (Version 0.14.1)). For multiple comparisons, one-way ANOVA and post hoc Tukey test for multiple comparison were used to calculate statistical significance. Pearson’s correlation was used to identify correlations between changes in $E_{m}$ measured under baseline and stretch states. ANCOVA test was used to compare the correlations obtained for fibrotic and healthy tissue. A *p* value < 0.05 was considered significant.

## Figures and Tables

**Table 1:** Parameters of a two-power law and estimated transition frequency. The parameters were fitted to data acquired for the fibrotic and healthy lungs in baseline and stretched. $f_{t}$ was derived from mean model parameters. Value for the high frequency exponent variable β was fixed at ¾ [31]. Values are shown as mean ± SE.

		A(kPa)	α	B(kPa)	$f_{t}$ (Hz)
β=3/4	healthy static	1.99±0.28	0.09±0.03	0.06±0.06	236
	healthy stretch	2.09±0.26	0.05±0.02	0.07±0.04	142
	fibrotic static	5.80±1.02	0.06±0.03	0.29±0.24	78
	fibrotic stretch	4.87±0.59	0.07±0.17	0.15±0.09	164

## Data Availability

Not applicable.
